# Use of Big Data and Machine Learning Methods in the Monitoring and Evaluation of Digital Health Programs in India: An Exploratory Protocol

**DOI:** 10.2196/11456

**Published:** 2019-05-24

**Authors:** Diwakar Mohan, Jean Juste Harrisson Bashingwa, Pierre Dane, Sara Chamberlain, Nicki Tiffin, Amnesty Lefevre

**Affiliations:** 1 Department of International Health Global Disease Epidemiology & Control Program Johns Hopkins Bloomberg School Public Health Baltimore, MD United States; 2 Faculty of Health Sciences Department of Integrative Biomedical Sciences, & Member of the Institute of Infectious Disease and Molecular Medicine University of Cape Town Cape Town South Africa; 3 Division of Epidemiology and Biostatistics School of Public Health and Family Medicine University of Cape Town Cape Town South Africa; 4 British Broadcasting Corporation Media Action New Delhi India; 5 Centre for Infectious Disease Research in Africa Institute of Infectious Disease and Molecular Medicine University of Cape Town Cape Town South Africa

**Keywords:** machine learning, mobile health, IVR messaging

## Abstract

**Background:**

Digital health programs, which encompass the subsectors of health information technology, mobile health, electronic health, telehealth, and telemedicine, have the potential to generate “big data.”

**Objective:**

Our aim is to evaluate two digital health programs in India—the maternal mobile messaging service (Kilkari) and the mobile training resource for frontline health workers (Mobile Academy). We illustrate possible applications of machine learning for public health practitioners that can be applied to generate evidence on program effectiveness and improve implementation. Kilkari is an outbound service that delivers weekly gestational age–appropriate audio messages about pregnancy, childbirth, and childcare directly to families on their mobile phones, starting from the second trimester of pregnancy until the child is one year old. Mobile Academy is an Interactive Voice Response audio training course for accredited social health activists (ASHAs) in India.

**Methods:**

Study participants include pregnant and postpartum women (Kilkari) as well as frontline health workers (Mobile Academy) across 13 states in India. Data elements are drawn from system-generated databases used in the routine implementation of programs to provide users with health information. We explain the structure and elements of the extracted data and the proposed process for their linkage. We then outline the various steps to be undertaken to evaluate and select final algorithms for identifying gaps in data quality, poor user performance, predictors for call receipt, user listening levels, and linkages between early listening and continued engagement.

**Results:**

The project has obtained the necessary approvals for the use of data in accordance with global standards for handling personal data. The results are expected to be published in August/September 2019.

**Conclusions:**

Rigorous evaluations of digital health programs are limited, and few have included applications of machine learning. By describing the steps to be undertaken in the application of machine learning approaches to the analysis of routine system-generated data, we aim to demystify the use of machine learning not only in evaluating digital health education programs but in improving their performance. Where articles on analysis offer an explanation of the final model selected, here we aim to emphasize the process, thereby illustrating to program implementors and evaluators with limited exposure to machine learning its relevance and potential use within the context of broader program implementation and evaluation.

**International Registered Report Identifier (IRRID):**

DERR1-10.2196/11456

## Introduction

Machine learning is an application of artificial intelligence that aims to allow computers to learn automatically from the analysis of large, highly granular datasets, with minimal human intervention [1]. In machine learning, models are created using existing data to make predictions about future events. Applications of machine learning in global public health are emerging, particularly in the context of digital health solutions, which have the potential to generate “big data.” Digital health encompasses the subsectors of health information technology, mobile health (mHealth), electronic health (eHealth), telehealth, and telemedicine.

Machine learning approaches in digital health have been mainly in the area of analyzing data generated by wearable sensors and accelerometers including efforts to predict and personalize monitoring systems for mobile patients [2], predict physical activity type and energy expenditure [3-5], and falls [6]. Beyond analyses of accelerometer data, studies have explored user engagement with different apps on mobile phones and tablets [7], and responses to patient feedback on health services [8]. Similar applications to social media data have sought to improve the detection of online illegal drug sales [9], depression [10,11], as well as explore vaccination sentiment trends and improve disease identification [12]. Analyses of geographic information system data have been used to map risk of exposure to disease [13]. Collectively these varied applications of machine learning have been classified by Mooney et al in three broad categories of (1) surveillance, including systems to monitor trends in disease incidence, health behaviors, and environmental conditions, (2) hypothesis-generating research, and (3) causal inference [1].

Evidence gathering on the effectiveness of digital health solutions is a growing field [14]. However, very few evaluations have sought to incorporate machine learning algorithms [8], and broader guidelines on the monitoring and evaluation of digital health solutions have stopped short of outlining options for predictive modeling [15]. Appropriate implementation of machine learning methodologies can facilitate dynamic, real-time interventions to improve data collection to assess program effectiveness and can also inform how data are used prospectively to improve program implementation.

In this paper, we outline methods proposed for the application of machine learning to the evaluation of two large-scale mHealth initiatives in India, which have scaled up to over 13 states in India since their initiation in 2012-2013. Kilkari is an outbound service that delivers weekly gestational age–appropriate audio messages about pregnancy, childbirth, and childcare directly to families on their mobile phones, starting from the second trimester of pregnancy until the child is one year old. Accredited social health activists (ASHA) mobilize women in the community to attend outreach and primary health center activities where auxiliary nurse midwives collect and register details of mothers and their pregnancies and, after delivery, children born in their catchment areas. Mobile Academy is an interactive voice response (IVR) audio training course for ASHAs in India. The training material delivered over the phone is designed to refresh their knowledge of life-saving preventative health behaviors and improve their interpersonal communications skills.

Through the use of these two digital health examples—the maternal mobile messaging service (Kilkari) and the mobile training resource for frontline health workers (Mobile Academy)—we illustrate possible applications of machine learning that can be applied to generate evidence on effectiveness, as well as to more broadly improve program implementation. We intend this paper to be an easy reference for public health practitioners considering the applicability of machine learning for digital health solutions, rather than a comprehensive review of the field of machine learning. In the course of the paper, we seek to provide an explanation in layman’s terms of the methods under consideration, for an audience unfamiliar with machine learning algorithms or advanced statistical methodologies. We also provide references throughout the text for further in-depth reading. Textbox 1 outlines the possible research questions that can be addressed by the study.

**Study aims.**

Aim 1: Describe subscriber losses at different points during pregnancy and postpartum period from the program

Objective 1a. Determine differences in subscribers who remain subscribed to Kilkari throughout the duration of service versus those lost at different points along the continuum of program databases.

Objective 1b. Develop a classifier for identifying different categories of losses.

Aim 2: Facilitate the program’s ability to identify and target ASHAs likely to perform poorly on digital health training programs and knowledge assessments

Objective 2a. Determine predictors of training course completion (overall and time to completion) by ASHAs based on performance on early modules of the course and other characteristics including reported motivation, knowledge, individual characteristics, and mobile literacy.

Objective 2b. Develop a classifier for the routine identification of ASHAs likely to perform poorly.

Aim 3: Understand the factors underpinning successful receipt of calls (are calls received?)

Objective 3a. Determine what proportion of calls successfully reach the end-user’s device for Kilkari.

Objective 3b. Identify the proportion of content specific to infant feeding and family planning received by end-users for Kilkari.

Aim 4. Determine how users are listening to messages

Objective 4a. Determine predictors for exposure to Kilkari content based on user characteristics.

Objective 4b. Measure exposure to Kilkari content based on technological and behavioral (end-user engagement) performance.

Objective 4c. Develop a classifier for measuring listening levels.

Aim 5: Understand optimal message delivery options for maximal impact

Objective 5a. Determine message delivery options with the most success in engaging client and assess patterns of listening.

Objective 5b. Determine the effect of early listening patterns (time of day of listening, duration and frequency of listening, content listening patterns) on postpartum engagement and overall exposure.

## Methods

### Summary

We present the methods section in parts. We first present a detailed description of the data we plan to use as our source including the architecture of the databases and data elements. Program data are currently held in different databases located in Gurugram and call data records are held in the Mobile Network Operator’s datacenter in Delhi. Next, we provide a description of the data munging (ie, data wrangling) and analysis methods including a brief description of the various machine algorithms under consideration.

### Data Sources and Flow

Auxiliary nurse midwives collect and register details of pregnant women and, after delivery, of postpartum women and children born in their catchment areas. These data are captured in print registers and uploaded at the block level by data entry operators, forming the data in the pregnancy tracking databases. The data collected include personal identifiers such as geographic location, names of women and a mandatory mobile phone number, and where available, details of the pregnancy and childbirth. Data capture happens at two key time points: (1) the earliest is the registration of the woman at the time of the identification of pregnancy, and (2) following childbirth, when the details for delivery care are available. In actual practice, these events may happen many days or months after the event (pregnancy registration or birth of child) has happened.

**Figure 1:**
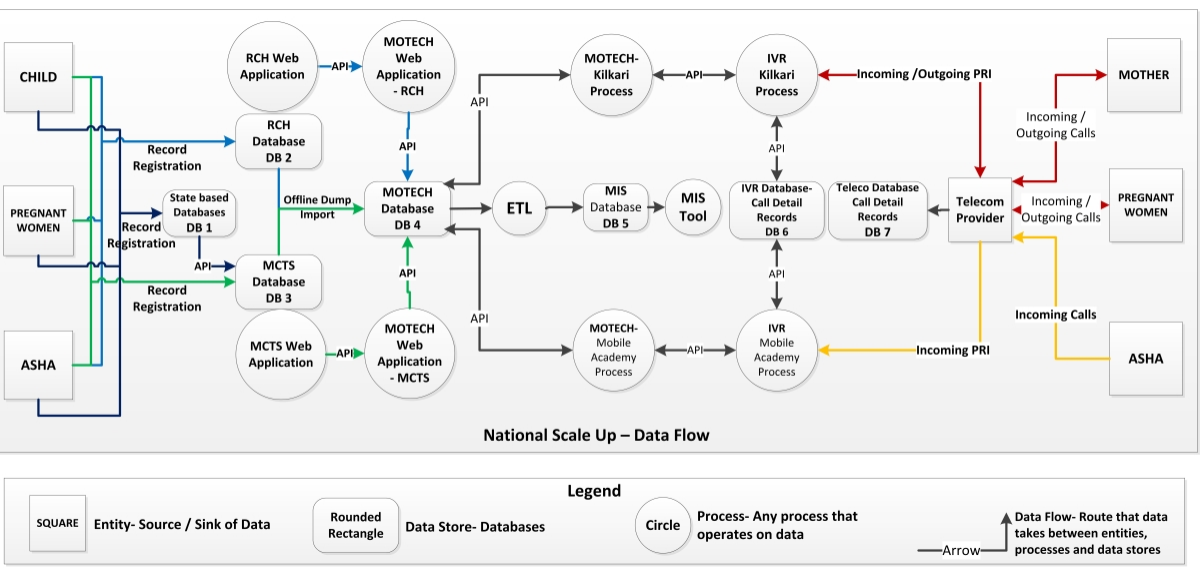
Summary of data flow for Kilkari and Mobile Academy.

Figure 1 summarizes the databases and flow of data for both Mobile Academy and Kilkari. The following are existing databases:

1. State-based databases that pre-date existing Mother-Child Tracking System (MCTS) and are integrated with MCTS
2. MCTS database at the national level
3. Reproductive Child Health (RCH) database at the national level
4. Call data records captured by Mobile Network Operator stored separately in their databases and used mainly for billing purposes
5. Call data records of Kilkari subscribers and ASHAs’ usage of Mobile Academy captured by the IVR system and stored in the IVR database in a data center contracted by the government
6. Mobile Technology for Community Health (MOTECH) database, which is integrated with the RCH and MCTS databases and integrates information from call data records with a small set of MCTS and RCH data for each user
7. Management Information System database, which extracts data from MOTECH and generates reports. The sampling frame for both Mobile Academy and Kilkari are derived from the MCTS and RCH data. Data from the RCH and MCTS databases are pulled into the MOTECH database on a predetermined schedule every day.

### Ethical Considerations

The registration data on pregnant women and ASHAs are collected by the Ministry of Health and Family Welfare of the Government of India and the ministries of health of the states participating in the program. The data will be analyzed under a data sharing agreement with the Bill & Melinda Gates Foundation and Johns Hopkins University, University of Cape Town, and BBC Media Action. The Institutional Review Boards of Johns Hopkins School of Public Health, Sigma in New Delhi, India, and the University of Cape Town have provided the ethical certification for the study.

### Data Processing

For the Kilkari program, the pregnant women or postpartum women’s data are captured in the RCH and MCTS systems, or in state-based systems that then pass data to RCH or MCTS, and from there to the MOTECH system. Before the data are accepted by MOTECH, the system automatically runs validations to check that the mobile numbers are in the correct format, locations match location masters in the MOTECH database, and last menstrual period and date of birth are within the Kilkari timeframe. The MOTECH system uses the last menstrual period or the delivery date to determine the schedule of messages to be delivered. The MOTECH engine provides the list of phone numbers (clients) to be called each day to the IVR system, which then calls the numbers and plays the appropriate pre-recorded message, which is stored in the IVR system’s content management system. If the call is not answered, then the IVR system attempts to call again at least 3 times every day for 4 days until the call is answered.

For the Mobile Academy program, details on ASHAs including their names, phone numbers, geographic location, and age are contained in either the RCH or MCT databases, or in state-based databases integrated with MCTS and used to register them to Mobile Academy. The MOTECH engine captures these data on ASHAs from the RCH or MCTS databases and following registration to Mobile Academy, ASHAs are eligible to call in to the IVR system using the same phone number provided in the RCH database. The IVR system validates the phone number against the MOTECH system and then retrieves the “bookmark” information that details the status of the ASHA and her progress on the list of content expected to be covered. Based on this information, the appropriate content is delivered to the ASHA via the IVR system and the updated data return to the MOTECH database.

### Analysis

The data from the databases (Figure 1) will be extracted onto secure password-protected hard drives from each server storage. Merging data files will be complex given the nature of identifiers across databases. An MCTS record does not have a beneficiary ID. Instead, it has a “Mother” (pregnancy) ID or a “Child” ID. In other words, MCTS tracks pregnancies and births, rather than women. When Kilkari first went live in October 2015, it mirrored the MCTS approach and generated subscription IDs for each pregnancy and then birth. However, the new RCH database does have a unique beneficiary ID, which enables the system to track an individual woman through her multiple pregnancies and the births of children. The architecture of the MOTECH database and Kilkari was changed in December 2016 to introduce a unique beneficiary ID and MOTECH was then integrated with RCH in mid-2017. There is an additional complexity, namely that MOTECH used to allow multiple Kilkari subscriptions on one mobile number, assuming a single phone could be shared by a number of women in a joint family. However, a decision was made to remove this feature in 2017 (July 28 for RCH and October 6 for MCTS) due to the complexity it created in analyzing system-generated data. Hence the analytic time horizon assumed in the analysis may span from 2017-2018 after the MCTS-RCH integration occurred and the aforementioned changes were made. The merging of datasets will occur in India, and only de-identified data will be stored on the hard drives and used in this analysis. As part of Study Aim 1, we will examine the quality of the data for completeness, including patterns and any geographic clustering in missingness.

### Additional Data From Baseline Surveys of Evaluation of Kilkari and Mobile Academy

Analyses described in this section are being carried out as part of a larger external evaluation of Kilkari. We describe concurrent efforts to undertake a randomized controlled trial (RCT) in the state of Madhya Pradesh (MP) for Kilkari, inclusive of baseline surveys with pregnant and postpartum women, and ASHA workers. Once identified as part of baseline survey activities and randomized to receive Kilkari content (or no content at all), phone numbers will be fed directly into the MOTECH database for provision of program services. For pregnant women, additional data collected as part of baseline household surveys include demographic factors (age, education, parity, literacy), socioeconomic characteristics (household assets, conditions), health care seeking and practices, as well as data on digital literacy and phone access. These data can be linked to MOTECH, IVR, and call center records to provide additional data elements. Overall, these data as well as data on technology performance (receipt of messages) and user engagement (behavioral performance) with content will help estimate exposure to Kilkari used in the assessment of causality as part of the RCT. For ASHAs, baseline survey data will include similar data elements on demographic, socioeconomic, and mobile literacy and phone access as well as knowledge and work-related variables linked to reported motivation and satisfaction. Overall, these added data elements can be linked to IVR and call record data for this subpopulation of Mobile Academy and Kilkari users in four districts of MP where the RCT is underway.

### Data Processing and Analysis

Descriptive statistics, including univariate plots like histograms, will be used to understand the distribution of each variable, including skewness and outliers. Multivariate plots like scatterplots and

locally weighted scatterplot smoothing (LOWESS) lines will be used to understand the relationships between different variables. Efforts to prepare the data are divided into two parts: splitting data into training and testing groups, and data processing.

#### Splitting Into Training and Testing

To avoid overfitting models that work well for the data in hand but fail to predict well with other datasets, the data will be split into three components. This is possible due to the large size of the dataset. The training set will comprise 60%, the test dataset 20%, and the validation dataset 20% of the data. The test set will be used to test and fine-tune the accuracy of predictive models, and the final selected model will be applied to the validation dataset. We anticipate having data from 2017, 2018, and 2019 and will ensure equal representation by random sampling. To ensure that the data are controlled for time as a confounder, subsets will be equally represented across different time periods.

#### Processing

Data processing is the act of preparing the data from its raw format into a usable format by the machine learning models. Indications for data processing will include (1) making the data easier to use; new indicators will need to be created to facilitate their use as predictors, (2) reducing computational cost of many algorithms by decreasing the number of variables, especially correlated and collinear variables, (3) removing noise due to outliers, and (4) making the results easier to understand.

### Algorithms

The most common methods by which algorithms learn about data to make predictions are supervised, unsupervised, and semisupervised learning [1]. Supervised learning trains algorithms using example input and output data, previously labeled by humans. Data may be labeled—a term used to denote that the outcome (or class) is known (eg, ASHA has completed the training module or not completed the training module)—or unlabeled. In contrast, unsupervised learning is concerned with uncovering structure and patterns within complex datasets based on information that is neither classified nor labeled. In unsupervised machine learning, the algorithms learn to infer structure based on unlabeled input data using clustering techniques. Semisupervised learning is a hybrid analytic technique, applied in contexts where the majority of data points are missing outcome information and yet prediction remains the goal [1].

In this program context, supervised machine learning algorithms are expected to be the primary analytic method employed because analyses are focused on classification using predictors and available data are expected to be labeled. The transformation of variables may be achieved by a variety of techniques including the creation of composite indicators and box-cox transformations. Unsupervised machine learning techniques, including dimensionality reduction techniques such as principal components analysis or K-means clustering, will be carried out as appropriate. Principal component analysis uses an orthogonal transformation to convert a set of observations of possibly correlated variables into a set of values of linearly uncorrelated variables called principal components. The first principal component has the largest possible variance and accounts for the highest proportion of the variance in the data, with each succeeding component accounting for the highest variance possible after accounting for the previous components. K-means clustering is a way to use data to uncover natural groupings within a heterogeneous population (Table 1). To uncover patterns, the algorithm starts by first assigning data points into random groups. The group centers are then calculated, and the group memberships are re-assigned based on the distances between each data point and the group centers. This process is repeated until there are no changes in the group memberships from the previous iteration [16]. In its application to Mobile Academy, K-means clustering will be used to detect patterns in ASHA engagement with training content, including training initiation and completion. Among Kilkari users, K-means clustering will be used to assess patterns in exposure to content by user characteristics based on data elements available in the RCH, including parity, age, and geographic area.

**Table 1:** Sample of data elements by source for Kilkari & Mobile Academy.

Database	Description	Sample of data elements anticipated for use in analyses	Variables	Boxes with reference to Figure 1
MCTS and RCH databases	National databases on reproductive, maternal newborn and child health care seeking among mothers and children <5 years of age Physically located in the NIC data center in Delhi	District, Mother & child unique IDs Pregnancy IDs ASHA IDs Last menstrual period Date of birth of child	Geographic identifiers Unique episode / beneficiary identifier	DB2, DB3
MOTECH database	Program database containing data on women and children as well as registered ASHAs Database works in conjunction with two algorithms, Kilkari and Mobile Academy, which function as “engines” for running the program Physically located in Railtel data center, Gurugram, Haryana	Kilkari: Activation date Deactivation date Calls answered Duration of content heard per message Mobile Academy: Course start date Course completion date Chapter wise scores Notification retry count Course passing date	Kilkari: Duration of enrollment in the program Status of enrollee Mobile Academy: Time taken to finish course Performance score of ASHA Number of tries by ASHA to complete modules	DB4
IVR database	Records when calls are triggered, what happens after the call is triggered (ie, does it get answered or does it fail, and if it fails why has it failed, eg, network errors, device errors) and whether the call needs to be retried; if yes, how many times. Records similar data for incoming calls. Physically located in Railtel data center, Gurugram, Haryana	Kilkari: Content file name Message duration Mobile Academy: Content identifier Duration of listening	Kilkari: Message identifier Duration of listening to message Mobile Academy: Content identifier Duration of listening	DB6
Call records database	Operator database on call handling Physically located in the Reliance data center, New Delhi	Call status Call failure reasons Call start time Call stop time Additional elements	Duration of listening to content	DB7

### Training of Algorithms

Once data have been processed, testing of algorithms will be carried out. Table 2 summarizes the algorithms proposed for training along with their intended applications to Mobile Academy and Kilkari. To determine the model with the best fit, we will explore several machine learning approaches in turn. Models will be fit on the training set, and the fitted model used to predict the responses for the observations in the validation set. The preferred analytic approaches will be selected based on their ability to minimize the total error of the classification, where the latter is defined as the probability that a solution will classify an object under the wrong category. We describe each approach considered below in lay terminology, along with indications for use, and its proposed application in the evaluations of Mobile Academy and Kilkari.

**Table 2:** Summary of algorithms proposed for testing and their intended application to Mobile Academy and Kilkari.

Algorithm		Description	Intended evaluation application	
			Mobile Academy	Kilkari
**Supervised**				
	1 Logistic regression	Classification (nonlinear model)	Classification of ASHA workers by user characteristics and patterns of training initiation by ASHAs	User characteristics associated with exposure to messaging content and duration in MP where face-to-face survey data have been collected
	2 Linear discriminant analysis	Classification (linear model). It is a linearization of Gaussian naïve Bayes.	Classification of ASHA workers by user characteristics and patterns of training initiation, completion, and performance	User characteristics associated with exposure to messaging content and duration
	3 Support vector machines (SVMs)	SVMs are techniques based on the calculation of the maximum margin hyperplane for the classification problems	Classification of ASHA workers by user characteristics and patterns of training initiation and completion	User characteristics associated with exposure to messaging content and duration
	4 Classification and regression trees	Predictive model that consists of leaves that represent the target and branches that represent conjunctions of inputs features. Considered a subset of decision trees. Random forests operate by constructing multiple decision trees during training and aggregating their results to avoid overfitting by single trees.	Classification of ASHA workers by user characteristics and patterns of training initiation and completion	User characteristics associated with exposure to messaging content and duration
	5 Naïve Bayes	Classification model based on probabilities	Classification of ASHA workers by user characteristics and patterns of training initiation and completion	User characteristics associated with exposure to messaging content and duration
	6 Neural Networks (NNs)	NNs are powerful models for machine learning. They are a generalization of linear and nonlinear models	Classification of ASHA workers by user characteristics and patterns of training initiation and completion	User characteristics associated with exposure to messaging content and duration
**Unsupervised**				
	7 K-means	K-means clustering is a way to use data to uncover natural groupings within a heterogeneous population	Grouping of ASHA workers by user characteristics and patterns of training initiation and completion	User characteristics associated with exposure to messaging content and duration

Our choice of methods will include a mix of algorithms based on their strengths and weaknesses and the objective of the process. A comprehensive comparison of supervised learning methods is provided in literature [17,18]. SVM and NNs are expected to perform better with continuous data while the Naïve Bayes method and decision trees perform better with discrete/categorical variables. Naïve Bayes and decision trees have good tolerance to missing values, while NNs and SVM do not. NNs and Naïve Bayes have difficulty handling irrelevant and redundant attributes (ie, extra variables with no useful information or variables with too many categories and too few numbers), while SVM and decision trees are insensitive towards them. Variables with high correlation negatively affect the performance of both Naïve Bayes and NNs, whereas SVM are relatively robust to correlated variables. While Naïve Bayes is robust to noise, NNs are sensitive to poor measurement of variables and susceptible to overfitting. NNs and SVM perform well with multidimensional data and when there is a nonlinear relationship between predictor and outcome. Naïve Bayes requires less memory for both training and validation phase, whereas NN requires large memory allocation across all phases. SVM and NNs usually outperform other methods while Naïve Bayes may yield less accurate results. Table 3 compares the strengths and weakness of different supervised machine learning methods.

### Testing and Validation

To facilitate decision making on the optimal analytic approach, three steps will be undertaken: (1) develop the correct model for each algorithm using the training dataset, (2) apply the final model for each algorithm on the test dataset, and (3) apply the best performing algorithm on the validation dataset.

In Step 1, algorithms will be run using the training dataset comprising 60% of the total sample from across all states for which data are available. For each algorithm, iterative testing will be run to select the best model that fits the data. The emerging results will then be assessed for model fit and accuracy. Table 4 summarizes the four proposed metrics for assessing the performance of each model.

**Table 3:** Performance comparisons of learning algorithms modified from Kotisiantis et al [17,18] (++++ represents the best and + the worst performance).

Model attributes	Decision trees	NNs	Naïve Bayes	SVM	Linear discriminant analysis	Logistic regression
Accuracy in general	++	+++	+	++++	+++	++
Speed of learning with respect to number of attributes and number of instances	+++	+	+++	+		
Speed of classification	++++	++++	++++	++++	++++	++
Tolerance to missing values	+++	+	++++	++	+++	+
Tolerance to irrelevant attribute	+++	+	++	++++	+++	+
Tolerance to redundant attributes	++	++	+	+++	++	+
Tolerance to highly interdependent attributes	++	+++	+	+++	++	+
Dealing with discrete/ binary/ continuous attributes	++++	+++ (not discrete)	+++ (not continuous)	+++ (not discrete)	++++	+++
Tolerance to noise	++	++	+++	++	+++	++
Dealing with danger of overfitting	++	+	+++	++	++	+
Attempts for incremental learning	++	+++	++++	++		
Explanation ability/ transparency of knowledge/ classification	++++	+	++++	+	+	+
Model parameter handing	+++	+	++++	+	++	+

**Table 4:** Metrics for assessing the performance of each model.

Model metrics	Formula^a^	Description
Accuracy	(TP+TN)/(TP+TN+FN)	Proportion of cases correctly classified
Precision or positive predictive value	TP/(TP+FP)	Fraction of relevant instances among the retrieved instances
Sensitivity or recall	TP/(TP+FN)	Fraction of relevant instances retrieved over total amount of relevant instances
Area under receiver operating characteristic curve (AUROC)	Area covered by the function of true positive rate/ false positive rate	The curve is plotted and area between the curve and the 45° line is multiplied by 2 to give the AUROC. A value of 1 represents perfect accuracy while 0.5 means the prediction is worthless.

^a^TP: true positive, TN: true negative, FP: false positive, FN: false negative

To illustrate the definition of performance metrics for Mobile Academy, we define true positives (TP) as the number of correctly classified ASHAs who have completed the training, and true negatives (TN) as the number of correctly classified ASHAs who have not completed the training. False positives (FP) are defined as the number of ASHAs incorrectly classified as having completed the training, while false negatives (FN) are the number of ASHAs incorrectly classified as not having completed the training.

Results from the performance metrics will help define the final model for each algorithm. In Step 2, these final models for each algorithm will be applied to the test dataset, which comprises approximately 20% of the total data. Using the same performance metrics, the models with the best fit and accuracy will be applied to the validation dataset as part of Step 3. Ultimately, predictions for Mobile Academy will aim to determine the probability of the ASHA finishing the course in a predetermined time frame and the possible score/performance of the individual ASHAs. For Kilkari, we will determine predictors for exposure to Kilkari content based on user characteristics, as well as explore the effect of early listening patterns on postpartum engagement and overall exposure.

## Results

The project has obtained the necessary approvals for the use of data in accordance with global standards for handling personal data. The results are expected to be published in August 2019.

## Discussion

### Principal Considerations

This paper presents the testing of a range of machine learning approaches to be incorporated as part of the evaluation of two large digital health programs in India—Mobile Academy and Kilkari. By utilizing machine learning approaches, we aim to improve the use of data for generating evidence on program reach and exposure as well as factors underpinning uptake for both programs. We will start by measuring dropped cases and missing data along the continuum of the databases. As part of this analysis, we aim to understand differentials in the characteristics of individuals lost along the continuum of databases and in turn, missed by the Mobile Academy or Kilkari programs. We then consider the technological performance as captured by four stakeholders: (1) government data systems (MCTS, RCH, webservices), (2) program’s call delivery and receipt systems, (3) mobile network operator (network coverage and quality), and (4) user device characteristics (switched on, within range of network). Finally, we explore user engagement with the program (behavioral performance) including patterns in the data that could predict key program performance metrics, including training completion for Mobile Academy and user coverage and exposure for Kilkari.

Analyses to measure dropped cases and missing data along the continuum of the databases, starting with the MCTS and RCH databases, are anticipated to generate insights into user relay of information to the Government of India including the provision of phone numbers by health workers and women. At present, there are no reliable estimates of the proportion of pregnant women with accurate mobile numbers, last menstrual periods, or dates of birth (for themselves or child) covered by the databases. More broadly, these analyses will help improve understanding of the quality of the RCH and MCTS data and underlying sampling frames used for Mobile Academy, Kilkari, and a range of other programs, including the characteristics of individuals included compared to those excluded. The predictors identified will help identify groups of women who are more likely to be missed by the program and ASHAs less likely to complete their course. These findings will also be used to inform the sampling of respondents for qualitative research to explore issues in depth and possibly identify opportunities for improving program targeting like registration of users.

Analyses to understand the technological performance of the program will build off of those conducted on the MOTECH platform in Ghana [19] and of MomConnect in South Africa [8]. In the former, IVR message delivery trends suggested that 25% or less of expected mobile health information messages were received by pregnant women [19]. While 20% delivery rates of successful outbound dialing calls are standard in the mobile industry, limitations in the timeliness of problem identification represent a missed opportunity for improving program exposure using, for example, call retry logics and systems. In South Africa, over 80% of short messaging service (SMS) messages were successfully delivered as part of MomConnect. SMS as a delivery channel has a much higher success rate than outbound dialing calls but suffers from other weaknesses in countries where illiteracy rates are high and local language fonts are not widely available on devices. In the case of MomConnect, however, challenges in the use of unstructured supplementary service data meant that 26% of initiated registrations did not convert into successful registrants [8]. These two examples reinforce the need to understand the user journey and follow the flow of data to understand whether the technology performs as intended.

Beyond understanding the technological performance of the program, and the wider telecommunications network and device landscape that it operates within, analyses will aim to measure user engagement, including predictors for exposure to content based on user characteristics. In the context of Kilkari, we will additionally plan to explore the effect of early listening patterns (eg, time of day of listening, duration and frequency of listening, content listening patterns) as a predictor for postpartum engagement and overall exposure. For Mobile Academy, we will use a mixture of unsupervised and supervised machine learning techniques to generate predictors of training course completion by ASHAs based on performance on early modules of the course. These will be externally validated using a range of data elements on other ASHA characteristics obtained from the broader evaluation, including reported motivation, knowledge, individual characteristics, and mobile literacy.

Overall, the proposed analyses are anticipated to complement primary data collection activities proposed as part of the summative evaluation of Kilkari and Mobile Academy. Findings emerging from this analysis will provide program implementers with tools for improving predictions of success and performance and provide insights into strategic use and collection of data. Elsewhere, deployments of similar programs including MomConnect in South Africa, Aponjan in Bangladesh, as well as other maternal messaging programs may be able to apply some of the same approaches described [8].

### Limitations

Ethical issues related to identifiers are important considerations for analyses described here. We will de-identify the databases at the point of data download, and all data will be secured in a password-protected hard storage device with access controlled by the Principal Investigator of the study. Once data are accessed, we note that findings will be only as reliable as the quality of underlying data. Completeness of the coverage of data and the data elements can be limited in low-resource settings. In recognition of this challenge, we will assess the quality of data for completeness and timeliness. The inherent design of the messaging program means that only those with mobile phones can be part of the sample, which could introduce selection bias in the association between outcome and predictors. Confounding will be an issue due to the many unmeasured variables associated with exposure such as mobile phone ownership, and registration into the pregnancy tracking database, and outcomes such as active listening to messages and adoption of healthy behaviors. Call answering does not automatically mean listening to the message by the intended client (eg, ASHAs or pregnant women) and as such, our analyses will be limited in its ability to identify the listener (information bias). However, the potential benefits of having other family members listen to information content could be immense. Beyond challenges with the measurement of exposure, the absence of a complete set of predictors will be present due to the limitations of such large-scale data gathering processes (incomplete model).

### Conclusions

This paper aims to provide a survey of approaches to the applications of machine learning to improve the implementation and evaluation of digital health programs. The two digital health examples described represent two of the largest digital health programs, based on number of active users, currently being implemented globally. Developing classifiers based on the above machine learning approaches will help identify gaps in data (Aim 1), target potential slow learners (Aim 2), measure exposure to program (Aim 3), characterize exposure levels in the population (Aim 4), and evaluate program impact (Aim 5). The scale of implementation and associated generation of data on user engagement with program content provides opportunities for big data analytics and more specifically, the use of machine learning approaches to improve the generation of evidence on program reach and exposure as well as factors underpinning uptake for both Mobile Academy and Kilkari.

## Abbreviations

ASHAs: accredited social health activists
IVR: Interactive Voice Response
MA: Mobile Academy
MCTS: Maternal and Child Tracking System
mHealth: mobile health
MP: Madhya Pradesh
NN: neural networks
RCH: reproductive child health
SVM: support vector machines
